# A novel classification to guide total hip arthroplasty for adult acetabular dysplasia

**DOI:** 10.3892/etm.2013.1093

**Published:** 2013-04-30

**Authors:** CHEN ZHU, MENG-QI CHENG, TAO CHENG, RUI-XIANG MA, RONG KONG, YONG-YUAN GUO, HUI QIN, SI FENG SHI, XIAN-LONG ZHANG

**Affiliations:** 1 Department of Orthopaedic Surgery, Shanghai Sixth People’s Hospital, Shanghai Jiao Tong University School of Medicine, Shanghai 200233;; 2 Department of Orthopaedic Surgery, Anhui Provincial Hospital, Anhui Medical University, Hefei, Anhui 230001, P.R. China

**Keywords:** adult, developmental dysplasia, total hip replacement, three-dimensional, computed tomography

## Abstract

In the field of hip arthroplasties, the secondary fixation of the implants depends directly on the quality of the primary stability. A good acetabular fit and metaphyseal filling between the prostheses and implants improve the initial stabilization, and optimize the transmission of forces to the bone. A precise knowledge of the three-dimensional acetabular or femoral shape is essential to the selection of adapted implants. A total of 63 patients diagnosed with developmental dysplasia were analyzed by three-dimensional computed tomography (3DCT), and the preoperative radiographic and 3DCT images were used to assess the acetabular/femoral deformities and variations of the hips. All joints were classified as Crowe type I, and bilateral measurements were taken for 10 patients. The acetabular abnormalities were classified according to the type of deficiency and the section angles of the acetabulum, with 26 hips (36%) classified as an anterior deficiency, 13 hips (18%) as a posterior deficiency and 34 hips (46%) as a lateral deficiency. The femoral side deformities were divided into three types according to the anteversion angle of the femur. A gradual increase in anteversion angle led to secondary rotational anomalies, and a narrowing of the canal at the isthmus. A total of 35 hips (48%) were classified as an F1 type deficiency, femur anteversion angle (FAVA) <30°; 32 hips (44%) as F2-type, 30°≤ FAVA ≤40°, with mild abnormalities of the femoral canal rotation and the diameter of the isthmus; and 6 hips (8%) as F3 type, FAVA >40°, with significant abnormalities of the femoral canal rotation and the diameter of the isthmus. This novel classification for adult acetabular dysplasia may provide a useful guide for surgery, and enable an improved selection of a suitable prosthesis.

## Introduction

Acetabular dysplasia (AD) is a developmental dysplasia of the hip (DDH), and is also known as hip joint instability. The characteristic pathological change in AD is a shallow acetabulum that leads to insufficient acetabular containment and coverage of the femoral head; however, radiographic observations have demonstrated that the femoral head remains in the true acetabulum (1). Studies from China have revealed that 50–60% of the patients who received a total hip arthroplasty (THA) suffered from osteoarthritis (OA) secondary to hip dysplasia, and a large number of adult AD patients ultimately undergo a total hip replacement (2,3). It has previously been suggested that the femoral and acetabular anatomical malformations that are apparent with AD increase gradually, in correlation with femoral head displacement (4). Since the patients with these anatomical malformations rarely develop further hip subluxations and dislocations, the majority of doctors do not consider the disorder to be a significant disability. However, anatomical variations of the acetabulum and proximal femoral medullary cavity are irregular (5), and preoperative X-rays do not identify all patients with AD; the correlation of the X-ray results with intraoperative findings varies greatly. A femoral neck fracture with AD is easily missed in clinical practice, and often leads to postoperative dislocation (6). The Crowe classification describes the proximal migration of the femoral head, regardless of the acetabular deformity, and assumes that there is a direct interrelation between the extent of the migration and the severity of disease (7). By contrast, the Hartofilakidis classification relies on the anatomy of the acetabulum, as encountered during surgery (8). However, the two classifications are not always valid, since the anatomy of the acetabulum and femur is variable, and the extent of migration is not a definite criterion for judging the type of dysplasia (8,9). Therefore, these classifications have limited uses as surgical guides, and for the selection of a suitable prosthesis. Furthermore, there is no specialized classification for mild DDH, such as AD.

With the increasing prevalence of THA, the incidence of adverse results, such as a fracture in the region surrounding the prosthesis and dislocation, has increased at follow-up. These adverse effects are often correlated with improper intraoperative management, most notably the implantation of a conventional prosthesis into an abnormal medullary cavity (10). The correct placement of a suitable prosthesis is the sole method of preventing adverse effects, and ensuring the long-term stability of the prosthesis. Thus, a more effective clinical classification is required to guide surgery. Following an analysis of previous studies, we propose, in the present study, a novel method of assessing acetabular and femoral deformities.

## Materials and methods

### Patients

From 2007 to 2011, 63 consecutive patients who were diagnosed with OA secondary to developmental dysplasia, or femoral neck fracture with developmental dysplasia, and who would accept a THA, were treated at Shanghai Sixth People’s Hospital (Shanghai, China). The patient cohort consisted of 14 males and 49 females, with a mean age of 55.6±12.5 years (range, 18–83 years). A total of 55 were diagnosed with OA, and eight with a femoral neck fracture. Patients who had undergone acetabular or femoral osteotomies or who suffered from rheumatoid arthritis were excluded from participation. In addition, patients in whom the dysplasia may have been affected by a neurological illness or Legg-Calvé-Perthes disease were also excluded. There were 32 cases of bilateral and 31 cases of unilateral AD. A total of 10 patients underwent a bilateral THA. Three-dimensional computed tomography (3DCT) was used to clarify whether a deformity existed and, if the result was positive, to identify the degree of acetabular or femoral deformity (11,12). A total of 30 acetabula or femurs were not able to be located in the normal anatomical sites, due to a significant acetabular or femoral deformity, out of 73 dysplastic hips. The study was conducted in accordance with the Declaration of Helsinki and with approval from the Ethics Committee of Shanghai Sixth People’s Hospital. Written informed consent was obtained from all participants.

### Radiographic evaluation

**Figure 1.**
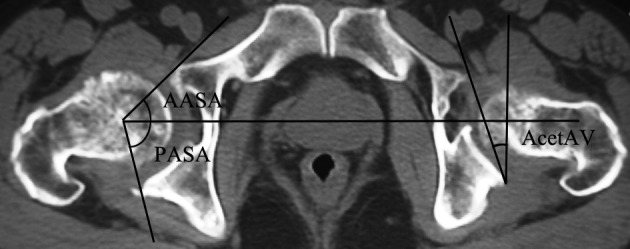
Reformatted axial image on which the acetabular anteversion angle (AcetAV), the anterior acetabular section angle (AASA) and the posterior acetabular section angle (PASA) passing through the center of the femoral heads were measured.

**Figure 2.**
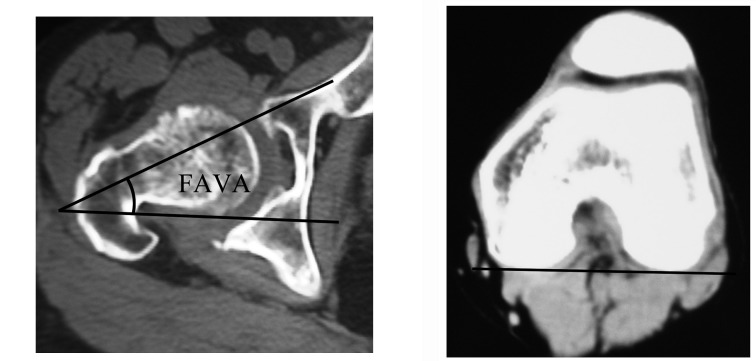
Femoral anteversion angle (FAVA) was defined as the angle between the femoral neck axis (A) and the transepicondylar axis (B).

**Figure 3.**
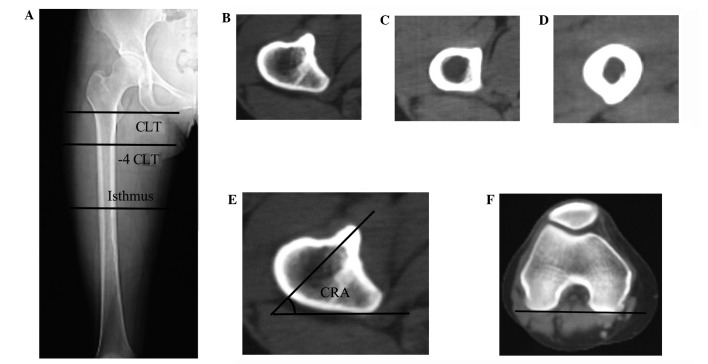
(A) Canal rotation angle (CRA) at three different cross sections of the femoral canal: (B) center of the lesser trochanter (CLT); (C) CLT-4 cm, 4 cm below the CLT; and (D) isthmus. (E) and (F) CRA: the angle between the major axis of the ellipses of best fit to the endosteal surface of the femoral canal and a tangent to the posterior aspect of the femoral condyles.

The radiographic evidence of AD included a central-edge angle of Wiberg (CE angle) <20° on the anteroposterior radiographs (13), and a Sharp angle >45° for the Crowe type I subluxation (14). In Crowe type I DDH, the vertical subluxation of the hip (measured from the inferior margin of the tear drop to the head-neck junction) is <50% of the diameter of the femoral head (or <10% of the height of the pelvis) (7). CT scans were acquired at a thickness of 1.2 mm, and a table speed of 3.0 mm/s, using a helical scanner (GE Lightspeed 16 Slice CT scanner, GE Healthcare, Waukesha, WI, USA). The helical scanning was conducted at 140 kVp and 300 mAs, and the field of view was 500 mm. Classifying the abnormalities using 3DCT involved basic scanning, ranging from 5 cm above the acetabular roof to the femoral condyles. The CT data were transferred digitally to Digital Imaging and Communications in Medicine (DICOM, version 3.0; National Electrical Manufacturers’ Association, Rosslyn, VA, USA), where the images were formatted (512×512 pixels), prior to the retrieval of the images using a compact disc or a digital versatile disc. These retrieved data were transferred to a personal laptop computer (IBM Lenovo Thinkpad X220i, Lenovo, Inc., Beijing, China), and the 3D bone images of the acetabulum and femur were reconstructed and analyzed using Intage Realia software (KGT, Inc., Tokyo, Japan). The original data were reconstructed in 1 mm intervals on coronal and sagittal images of the hip joint (12). Two experienced hip surgeons, who were responsible for performing >200 cases each year, subsequently measured the following parameters, twice (8): i) Anterior acetabular section angle (AASA), i.e. the angle between the centerline extending between the bilateral femoral heads, and the line from the center of the head to the anterior margin of the acetabulum (59–83° and 53–92° in normal males and females, respectively (12); Fig. 1); ii) posterior acetabular section angle (PASA), i.e. the angle between the centerline extending between the bilateral femoral heads, and the line from the center of the head to the posterior margin of the acetabulum [84–116° and 87–120° in normal males and females, respectively (12); Fig. 1)]; iii) acetabular anteversion angle (AcetAV), i.e. the angle between the line extending between the anterior and posterior margins of the acetabulum, and the line perpendicular to the center line connecting the bilateral femoral heads (12) (Fig. 1); iii) femur anteversion angle (FAVA) (15) (Fig. 2); iv) canal rotation angle (CRA), i.e. the angle between the major axis of the ellipses of best fit to the endosteal surface of the femoral canal, and a tangent to the posterior aspect of the femoral condyles (4,11) (Fig. 3); v) medio-lateral and vi) antero-posterior canal width at the level of the canal isthmus (the maximum value of the medio-lateral or antero-posterior extracortical diameter of the diaphysis was also recorded); and vii) canal diameter at the isthmus (the point of the medullary canal with the smallest cross-sectional area). The mean of the normal population was used as the control (4,11). Forty-six healthy controls with normal hip anatomy were also assessed, including 11 males and 35 females, with a mean age of 56.7±11.7 years (range, 25–80 years).

### Classification

The acetabular abnormalities were classified into A1-type anterior, A2-type posterior and A3-type lateral (including mild and global) deficiencies (Table I) (12). The femoral classification was as follows: F1-type, FAVA <30°; F2-type, 30°≤ FAVA ≤40°, with mild abnormalities of the femoral canal rotation and the diameter at the isthmus; F3-type, FAVA >40°, with significant abnormalities of the femoral canal rotation and the diameter at the isthmus (Tables II and III). There were 21 A1-type cases (26 hips), nine A2-type cases (13 hips) and 33 A3-type cases (34 hips). In addition, there were 33 F1-type cases (35 hips), 26 F2-type cases (32 hips) and four F3-type cases (six hips).

### Statistical analysis

The database was established via statistical analysis using SPSS 19.0 (SPSS, Inc., Chicago, IL, USA). For variables that were normally distributed, differences between the types were evaluated using analysis of variance (ANOVA), followed by the unpaired t-test for multiple pair-wise comparisons of all significant variables. Categorical data were compared using the χ^2^ test. To assess the intraobserver reliability of the different parameters of the femur or the acetabulum, the preoperative radiographs for each patient were templated by an investigator, who subsequently repeated the templating two weeks later. In addition, the templating procedure was repeated by a second investigator, independently. The intra- and interobserver effects were calculated using an intraclass correlation coefficient (ICC) (8). Pearson’s correlation coefficient was used to assess the correlations between various measurements. P<0.05 was considered to indicate a statistically significant difference (Tables II and III).

## Results

When the acetabular and femoral abnormalities were divided into subgroups, using 3DCT, it was observed that there was a crossover between each of the femoral subtypes (F1, F2 and F3) and the acetabular subtypes (A1, A2, or A3), with the exception that the F3-type deficiency did not appear in conjunction with the A2-type deficiency. Significant differences were demonstrated in the AcetAV (P<0.05), AASA (P<0.05) and PASA (P<0.05) between the A1, A2 and A3-type deficiencies (A1 versus A2, A1 versus A3 and A2 versus A3); however, no significant differences were observed in the CE angle (P>0.05) or the Sharp angle (P>0.05). The AASA values of the A1, A2 and A3-type deficiencies were significantly different from that of the control group (P<0.05), whereas only the PASA values of the A2 and A3-type deficiencies were significantly different in comparison with the PASA value of the control group (P<0.05; Table II). There was a significant Pearson’s correlation between the AASA and the AcetAV (r=−0.353, P=0.002), and between the PASA and the AcetAV (r= 0.5, P= 0.001), indicating that hips with a greater AASA also had a lower AcetAV, and that those with a greater PASA also had a higher AcetAV. No significant differences were observed in the AcetAV between the A3-type deficiency and the control (t=0.102, P=0.92). The intra- and interobserver reliability values of the acetabular classification, obtained using ICC, were 0.843 and 0.862, respectively, which indicated good reproducibility in the acetabular measurements.

Table III displays the canal width at the level of the isthmus in the antero-posterior and medio-lateral directions, and the canal diameter at the isthmus; significant differences were observed between the control and the F2 and F3-type deficiencies (P<0.05), but not between the control and the F1-type deficiency (P>0.05). There was no significant difference in the canal diameter at the isthmus between the F2 and F3-type deficiencies (P=0.336), although the mean diameter of the canal of the F3-type femurs was smaller than that of the F2-type femurs (8.9 mm versus 9.7 mm). The CRAs at the three levels were significantly different between the F2 and F3-type deficiencies (P<0.05). There were no significant differences in the CRAs between the F1-type deficiency and the control cases; however, significant differences were observed in a comparison between the F2 and F3-type deficiencies and the control (P<0.05). From the center of the lesser trochanter (CLT) to the medullary cavity of the isthmus, a gradual increase was observed in the CRA. However, it was observed that there was a significantly higher mean increase in the CRA from the CLT to the isthmus in the control cases (40°), in comparison with that of the F2 (34°) and F3 (28°)-type deficiencies. The variation in femoral anteversion in the F3-type deficiency was of a greater significance than that in the F1 and F2-type hips (P<0.05). It was observed that femurs with a greater FAVA also appeared to have narrower canals (r=−0.315, P=0.007), and a smaller CRA at the isthmus (r=−0.696, P= 0.007).

There was no significant correlation between the FAVA and the AcetAV in the dysplastic hips overall (r=0.001, P=0.996). However, when the hips were divided into subgroups, a significant positive correlation was observed between the FAVA and the AcetAV in the anterior deficiency subgroups (r= 0.394, P= 0.046). By contrast, there was no significant correlation between the FAVA and the AcetAV in the posterior and global deficiency subgroups (r=−0.006, P=0.973; and r=0.038, P=0.829, respectively). The intra- and interobserver reliability values of the femoral classification, obtained using ICC, were 0.813 and 0.822, respectively, which indicated that there was an acceptable reliability in the femoral measurements. There were no significant differences in the average age (t=0.585, P= 0.561) or gender (χ^2^= 0.040, P= 0.836) of the 63 patients with AD compared with the 46 healthy controls. In the control hips, no significant correlations were observed between the FAVA and the AcetAV, Sharp angle or CE angle (r=−0.115, P= 0.448; r= 0.041, P= 0.785; and r= 0.026, P= 0.078, respectively). However, there was a significant positive correlation between the FAVA and the Sharp angle (r=0.456, P=0.00), and a significant negative correlation between the FAVA and the CE angle (r=−0.473, P=0.00) in the dysplastic hips.

## Discussion

In this study of 73 dysplastic hips and 46 normal hips, the morphological differences between dysplastic and normal hips were observed, and significant correlations between the AcetAV and the acetabular anterior or posterior deficiency subgroups were identified. In addition, it was demonstrated that there was a significant correlation betwen the femoral anteversion and the AcetAV in the anterior deficiency subgroup. It was revealed by Akiyama *et al* (5) that changes in the AASA, PASA and AcetAV may be detected by 3DCT, and that 3DCT clearly exhibits the location and extent of the dysplasia. In a study by Ito *et al* (12), 22 of 84 AD hips (26%) were classified as having an anterior deficiency; 17 (20%), a posterior deficiency; and 45 (54%), a lateral deficiency. Hips with poor anterior acetabular support were defined as those with an AASA <50°, while hips with poor posterior support were defined as those with a PASA <90° (12). In a previous study, the AASA, PASA, and AcetAV measurements were demonstrated to be effective for the precise evaluation of various acetabular deficiencies (16). Anda *et al* (17) revealed that the AcetAV in the anterior deficiency subgroup was significantly larger than in the other groups. By contrast, the AcetAV in the posterior deficiency subgroup has been observed to be smaller than that in the normal and global deficiency subgroups (5). The results of these studies supported the observations in the present study. In addition, the results of the present study demonstrated a trend towards increased or decreased acetabular anteversion in shallow hips with poor anterior or posterior support.

The previously mentioned results indicated the existence of a potential developmental interaction between the femur and acetabulum. When the dysplastic hips were divided according to the location of the acetabular bone defect, significant differences in acetabular version were observed among the subgroups. It was demonstrated that hips with a larger FAVA appeared to additionally have an increased AcetAV, indicating a biomechanical cycle resulting in the pathology of dysplastic hips with anterior acetabular deficiency (5). By contrast, no correlation in version was observed in hips with a posterior or global deficiency. However, it was demonstrated that there was a significant correlation between the FAVA and the Sharp/CE angles in the dysplastic acetabula.

Although the previous studies indicated that each type of dysplasia was correlated with the degree of the dysplasia, rather than the specific type of severity, they did not offer a systematic and detailed guide for THA. The large individual acetabular morphological variability across all levels of dysplasia observed in this study demonstrated that it is not possible to select an acetabular prosthesis for dysplastic hips on the basis of the severity of the subluxation alone. The results of the study suggested that there is a requirement for the surgeon to choose the type of socket implantation according to the type and extent of the acetabular defect, and to adapt to the individual FAVA. Thus, it is necessary for each patient be considered individually, in order that the angle of the acetabular cup may be customized to suit (9). For the A1-type deficiency, a reduction in the AcetAV or a neutral position is required when the cup is implanted. In cases with an excessive FAVA, a decrease in the FAVA is required for the inclusion and congruity of the hip joint (5). For the A2-type deficiency, an appropriate increase in the AcetAV is required to resolve the initial instability, in order to prevent the aggravation of the posterior acetabular insufficiency. In the present study, no significant differences were found in the AcetAV between the A3-type deficiency (mild or global) and the control group. The acetabular defects predominantly occurred on the upper and lateral margins of the acetabulum, although anterior and posterior deficiencies were also observed with the global deficiency. Due to the absence of structural bone defects in the acetabula, and since the acetabular cup covers >70% of the bone bed, there is a requirement for the acetabular cup to be placed at the center of the acetabulum, and for normal anteversion to be maintained (2). Since mild or global deficiencies of the acetabulum require similar methods of prosthetic implantation, hips with these types of deficiency may be classified as having a lateral deficiency (12). The treatment of femoral abnormalities or variations with A1, A2 or A3-type deficiencies are described in greater detail later in this study.

The present study revealed the morphological characteristics of dysplastic femurs, and investigated the effects of the disorder on the geometry of the intramedullary canal. These results were then compared with a control group. It was demonstrated that in cases with excessive anteversion, the dysplastic femurs were smaller than the control femurs, with narrower, straighter and less-tapered canals. Sugano *et al* and Noble *et al* (4,11) observed that the 3D anatomy of a femur with the mildest degree of subluxation (Crowe type I) exhibited a significantly different FAVA and medullary cavity rotation, and that several patients had a FAVA >60°. In addition, it was demonstrated that the diameter of the femoral medullary cavity was reduced in the Crowe type I femurs. The minimum diameter of the canal in the Crowe type I femurs was 1 mm less than in the control femurs (4,11). Argenson *et al* (9) revealed that the mean diameter of the medullary cavity was >1.6 mm narrower in the antero-posterior and >1.9 mm narrower in the medio-lateral position in the Crowe type I than in the control femurs (9,11). The canal flare and metaphyseal canal flare indices were used to assess variations in the width of the femoral medullary cavity on anteroposterior radiographs (18). X-rays are only able to assess the femoral marrow cavity in two dimensions; however, with the exception of the differences in canal width, it is important that the morphological characteristics of the femoral medullary cavity at different levels are identifiable with 3DCT. Therefore, the measurements were performed in three dimensions, i.e. in the axial, coronal and sagittal planes (19). It was observed that the normal rotation angle of the medullary canal gradually increased from the CLT to the isthmus. However, in the dysplastic femurs, the increased ante-version of the proximal femur resulted in a reduction in the rotation in the medullary canal, predominantly in the region from the CLT to the canal at the isthmus (4,11). The variation between the F2 and F3-type deficiencies supported this observation. With regard to surgery, this variation is critical, since it is necessary to be aware of variations in the width of the medullary cavity when the femoral canal is reamed, in order to avoid femoral fractures. When the femoral stem is implanted, there is a requirement for the morphological differences that occur at different levels of the medullary cavity to be considered, in order to ensure that the prosthesis closely matches the medullary cavity of the femur. Therefore, when the FAVA is exaggerated, the rotational orientation has a marked effect on the size and shape of the canal (11), and the concomitant twist of the femoral canal increases the difficulty of the joint replacement.

The results of the present study demonstrated that the position of the femoral anterior arch in the femurs with AD was not significantly different from that observed in the control group. This indicated that the primary anatomical feature affecting the successful placement of the stem is increased femur anteversion, leading to secondary rotational anomalies and a narrowing of the canal at the isthmus. Therefore, these features were the basis of our classification (4,9). With regard to the F1-type deficiency (FAVA <30°), femoral stem implantation with a normal FAVA is possible. For the F2-type deficiency (30°≤ FAVA <40°, with mild abnormalities of the femoral canal rotation and the isthmus diameter) it may be appropriate to adjust the FAVA from 15° to 25°, due to the anteversion of the acetabular cup. However, following femoral neck osteotomy, the cross-section of the long axis of the femur is not usually consistent with that required by the femoral stem. If a proximal fixed prosthesis is chosen, stability is poor; therefore, in the majority of cases a prosthesis with a straight and thin distal stem is required to accommodate this diaphyseal femoral anatomy (20). With regard to F3-type deficiencies, with significant abnormalities of the femoral canal rotation and a reduced isthmus diameter, it has been demonstrated that modular or customized components are necessary, in order to accommodate the shape of these dysplastic canals (21,22). Furthermore, the present study indicated that the templating technique exhibited the desired reliability, with all the ICC values exceeding 0.8 (8).

A retrospective database and image review was used to summarize the diversity of mild dysplasia; this reinforced the observations of a number of previous studies, concerning the exaggerated anteversion in mildly dysplastic femurs. At present, the majority of doctors do not consider the disorder of mild dysplasia to be a great disability, and, furthermore, preoperative 3DCT scans are not routinely requested for Crowe type I hips, due to the additional medical expense. However, the present study revealed the anatomical variations of the acetabulum and proximal femoral medullary cavity to be irregular and interrelated (∼41.1% of cases), and preoperative X-rays and 2DCT scans of the hip joint are not able to identify any correlation between these variations. It is therefore important that the results of 3D scans are assessed preoperatively, and that any interrelation between the femoral and acetabular morphologies is identified by the surgeons. The aim of this investigation was to emphasize the morphological variations in mild dysplasia, particularly in the femoral medullary cavity and the acetabulum, as a primary step to determining the potential requirements for surgical procedures. The results of this study are likely to provide a greater insight into the morphological characteristics of dysplastic hips, and the challenges confronting joint replacement surgeons.

In addition to suggesting a novel anatomic classification, this study provided a detailed characterization of the anatomical variations to be considered in hip arthroplasty implants for patients with AD. The purpose of this 3D morphometric analysis was to serve as an anatomical reference for acetabular and femoral implants. The novel classification employed in this study used 3DCT measurements to clarify the location and extent of acetabular deficiency, the diameter of the medullary cavity at the isthmus and the degree of rotational deformity. This is likely to facilitate the improved management of malformations of the acetabulum and femur, and to ensure the selection of a suitable prosthesis. Since the initial assessment of the patients with AD has been adopted, the individualized prosthesis implantation and surrounding bone matching have achieved the desired results, thereby increasing the long-term survival rates of the prostheses.

## Figures and Tables

**Table I.** Classification of acetabular dysplasia.

Parameter	A1-type anterior deficiency	A2-type posterior deficiency	A3-type lateral deficiency	
			Mild deficiency	Global deficiency
AASA	<50°	≥50°	≥50°	<50°
PASA	≥90°	<90°	≥90°	<90°

AASA, anterior acetabular section angle; PASA, posterior acetabular section angle.

**Table II.** Comparison of computed tomography measurements among the different types of acetabular deficiency.

Group	n	CE angle (°)	Sharp angle (°)	AcetAV (°)	AASA (°)	PASA (°)
A1-type	26	12.7±7.1^a^	50.2±3.1^a^	22.5±1.8^a^	48.3±2.4^a^	93.3±6.0
A2-type	13	13.5±4.2^a^	49.9±4.3^a^	14.0±3.4^a^	60.2±3.1^a^	78.2±4.0^a^
A3-type	34	11.9±5.7^a^	52.1±5.0^a^	19.6±4.6	54.6±8.5^a^	88.4±10.1^a^
Control	46	31.0±4.3	35.9±2.9	19.8±3.7	75.9±8.6	95.3±6.0

Data are presented as the mean ± standard deviation.

a P<0.05 compared with control. CE angle, central-edge angle of Wiberg; AcetAV, acetabular anteversion angle; AASA, anterior acetabular section angle; PASA, posterior acetabular section angle.

**Table III.** Anatomical parameters of control and dysplastic femurs, based on the different types of acetabular dysplasia.

Parameters	Control (n=46)	F1-type (n=35)	F2-type (n=32)	F3-type (n=6)
Medio-lateral canal width at isthmus (mm)	12.4±1.4	12.3±1.5	11.7±1.3^a^	11.1±0.5^a^
Antero-posterior canal width at isthmus (mm)	13.6±1.4	13.4±1.6	12.8±1.3^a^	12.2±0.7^a^
Canal diameter at isthmus (mm)	10.3±1.4	10.4±1.7	9.7±1.2^a^	8.9±0.4^a^
Canal rotation angle (°)				
At CLT	45.2±3.7	46.4±2.7	48.6±2.0^a^	52.1±2.1^b^
CLT-4 cm	49.8±3.4	50.7±2.7	54.0±3.2^a^	58.6±1.1^b^
At isthmus	85.2±3.6	84.1±2.5	82.4±1.6^a^	79.8±1.8^b^
FAVA(°)	18.6±5.0	25.8±1.5^a^	32.2±2.5^a^	45.0±3.7^b^

Data are presented as the mean ± standard deviation.

a P<0.05 compared with control;

b P<0.05 compared with F2-type deficiency. F1-type, femur anteversion angle (FAVA) <30°; F2-type, FAVA ≤40°; F3-type, FAVA >40°. CLT, center of the lesser trochanter.
